# Focal dystonia in musicians: linking motor symptoms to somatosensory dysfunction

**DOI:** 10.3389/fnhum.2013.00297

**Published:** 2013-06-25

**Authors:** Jürgen Konczak, Giovanni Abbruzzese

**Affiliations:** ^1^Human Sensorimotor Control Laboratory, Center for Clinical Movement Science, School of Kinesiology, University of MinnesotaMinneapolis, MN, USA; ^2^Departments of Neuroscience, Rehabilitation, Ophthalmology, Genetics and Maternal Child Health, University of GenoaGenoa, Italy

**Keywords:** human, motor control, movement disorder, proprioception, sensorimotor integration, sensory integration

## Abstract

Musician's dystonia (MD) is a neurological motor disorder characterized by involuntary contractions of those muscles involved in the play of a musical instrument. It is task-specific and initially only impairs the voluntary control of highly practiced musical motor skills. MD can lead to a severe decrement in a musician's ability to perform. While the etiology and the neurological pathomechanism of the disease remain unknown, it is known that MD like others forms of focal dystonia is associated with somatosensory deficits, specifically a decreased precision of tactile and proprioceptive perception. The sensory component of the disease becomes also evident by the patients' use of “sensory tricks” such as touching dystonic muscles to alleviate motor symptoms. The central premise of this paper is that the motor symptoms of MD have a somatosensory origin and are not fully explained as a problem of motor execution. We outline how altered proprioceptive feedback ultimately leads to a loss of voluntary motor control and propose two scenarios that explain why sensory tricks are effective. They are effective, because the sensorimotor system either recruits neural resources normally involved in tactile-proprioceptive (sensory) integration, or utilizes a fully functioning motor efference copy mechanism to align experienced with expected sensory feedback. We argue that an enhanced understanding of how a primary sensory deficit interacts with mechanisms of sensorimotor integration in MD provides helpful insights for the design of more effective behavioral therapies.

## Introduction

**Figure 1:**
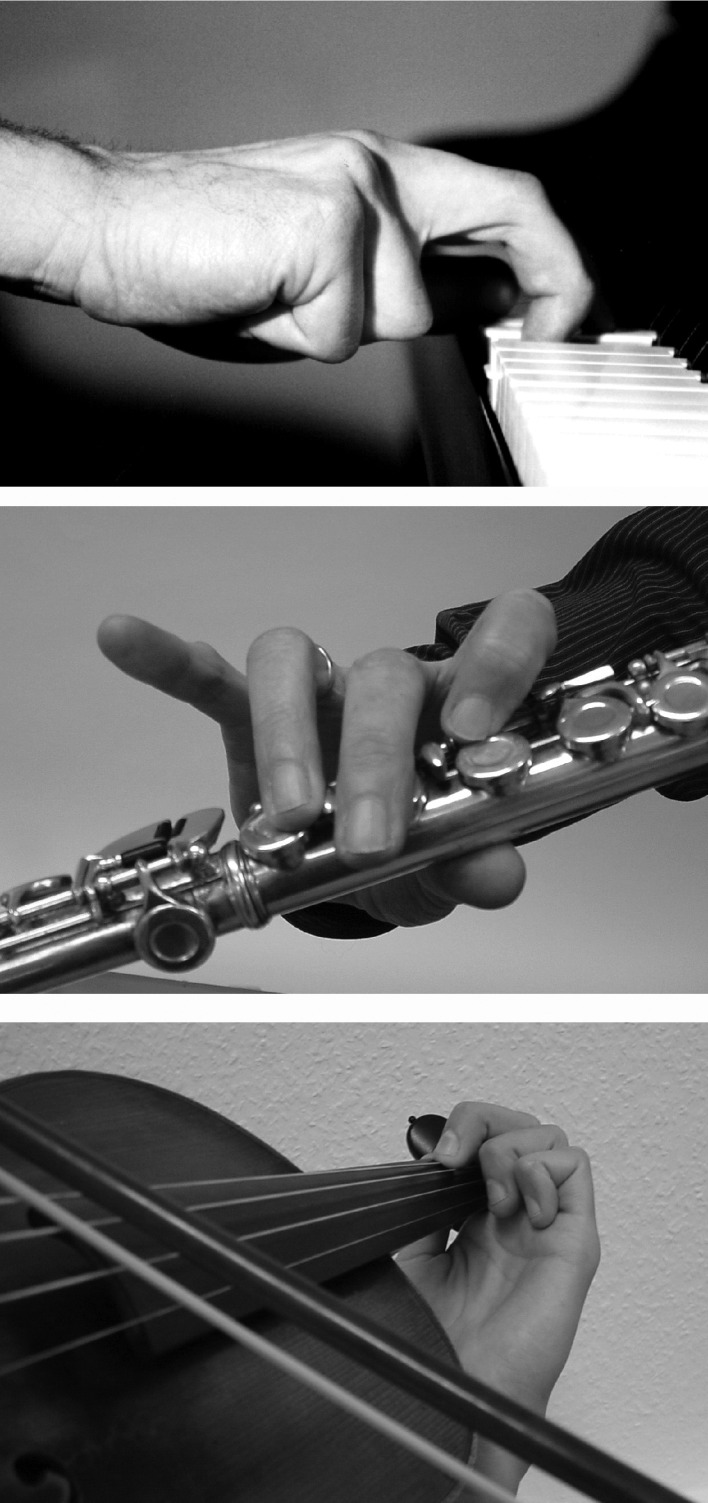
**Typical patterns of dystonic postures in a pianist, a violinist, a flutist**. Note the involuntary flexion of the ring and little fingers (digits 4 and 5) in the pianist and violinist. The flutist's index finger is involuntarily flexed while middle, ring and little fingers attempt to compensate by extending.

Musician's dystonia (MD) is a motor disorder consisting of involuntary sustained muscle contractions that interfere with the voluntary motor control during the play of a musical instrument. It is task-specific because the dystonic movements are primarily observed during the execution of a confined set of motor actions associated with musical play, while other movements of the same motor system remain intact. Several others forms of task-specific dystonia are known. Collectively, they have been termed “occupational cramps” because they usually affect distinct acquired sensorimotor skills, such as writing, putting in golf, or the playing of a musical instrument (Rosenbaum and Jankovic, 1988; Jankovic and Ashoori, 2008). Musicians are said to be at particular risk for dystonia, especially pianists, guitarists and woodwind players (Zeuner and Molloy, 2008). In patients with MD the loss of control often manifests itself during fast passages, the irregularity of trills, or involuntary flexion of one or more fingers (Altenmüller, 2003) (see Figure 1). The loss of synergistic muscle control also becomes apparent as a cocontraction of antagonistic muscle groups. For example, in pianist's cramp, the coactivation of wrist flexor and wrist extensor muscles is frequently observed. MD symptoms can become so severe that the musician is forced to give up playing the instrument effectively terminating a professional career. The prevalence of MD is estimated to be approximately one percent of all professional musicians (Altenmüller, 2003; Altenmüller et al., 2012). While the underlying pathophysiology of MD and other forms of focal dystonia remains unclear, it has long been assumed to involve abnormal functioning of the basal ganglia that ultimately affect the sensorimotor cortices. However, the exact link between the observable dystonic symptoms and the neural dysfunction in the basal ganglia is not yet established.

This paper is concerned with the sensory aspects of MD, especially those related to touch and proprioception. In light of the recent research on sensory deficits associated with dystonia and other basal-ganglia related diseases, we will present a sensorimotor systems framework that is based on computational models of motor control and seeks to explain the origin of observed dystonic symptoms. While such framework is not suitable to explain the underlying pathological mechanisms at a neuronal level, it is well suited to better understand the failed and compensatory sensorimotor control aspects of the disease. Such enhanced understanding may spawn new behavioral therapeutic interventions for a disease that currently can be treated with varying success (Schuele et al., 2005) but cannot be cured by pharmacological means.

## Evidence for somatosensory deficits in focal dystonia

Next to the task-specific forms of dystonia such as MD or writer's cramp, other types of focal dystonia are not task-specific. That is, specific muscle groups are generally affected, such as the neck musculature in cervical dystonia or eye and facial muscles in blepharospasm. Classically, all these forms of primary focal dystonia have been considered to be pure “motor” disorders where the final organization and execution of movement is affected. However, today it is also firmly established that a number of “non-motor” abnormalities including sensory and cognitive aspects of motor control, depression and sleep problems (Avanzino et al., 2010) can be associated with focal dystonia (for a review see Stamelou et al., 2012). It is not uncommon for patients with focal dystonia to complain of mild sensory symptoms such as pain or discomfort in the affected area before dystonic symptoms develop (Ghika et al., 1993; Martino et al., 2005), while conventional clinical sensory examination is usually normal.

There are numerous research reports documenting that patients with focal dystonia have an impaired ability in discriminating tactile stimuli in both the spatial and temporal domain (Tinazzi et al., 1999; Bara-Jimenez et al., 2000; Scontrini et al., 2009). Abnormalities in the temporal perception of consecutive tactile stimuli have been shown in unaffected carriers of the DYT1 mutation (Fiorio et al., 2007) suggesting that such abnormality may precede the overt manifestations of dystonia and in psychogenic dystonia (Morgante et al., 2011). Moreover, proprioceptive-based finger position sense thresholds and the perception of arm motion have been reported to be abnormal in patients with cervical dystonia or blepharospasm (Grünewald et al., 1997; Putzki et al., 2006). The abnormalities in both tactile and proprioceptive processing are not restricted to the affected dystonic musculature, but were also documented in non-affected body regions (Molloy et al., 2003; Putzki et al., 2006; Fiorio et al., 2008). Altogether these findings indicate a generalized somatosensory deficit in focal dystonia where the abnormalities of detecting and discriminating somatosensory stimuli represent a widespread neurophysiological trait.

Such loss in the sensitivity and acuity of the tactile and proprioceptive senses ultimately affects a dystonic person's body scheme. This has been confirmed by experiments that involve mental simulation and prediction of bodily movement. For instance, mental rotation of body parts was found defective in both the affected and unaffected hand of patients with writer's cramp (Fiorio et al., 2006). Another way to investigate someone's sense of body ownership is the so-called “rubber hand paradigm.” In this paradigm an illusion of ownership is established by synchronous stroking of the participants' real unseen hand and a visible fake hand, whereas similar asynchronous stroking does not illicit the illusion. Normally, a drift in the perception of the real hand toward the rubber hand is expected only after synchronous stroking. However, in patients with focal hand dystonia this proprioceptive drift was disrupted in the dystonic hand while the subjective experience of the illusion was retained (Fiorio et al., 2011). This finding is consistent with the notion that focal dystonia can lead to a dissociation between a person's body scheme and the body-relevant sensory information signal (here the afferent proprioceptive signals indicating a specific hand position). Such altered body scheme in focal dystonia is likely not a primary symptom of the disease, but results from the interaction between a stored neural representations of the body and limb system and failed processes of sensorimotor integration. We will highlight this issue in more detail below.

## Neurophysiological correlates of focal dystonia

The exact cause of the deficits in the processing of tactile and proprioceptive afferents is not known. However, the results of numerous studies recording somatosensory evoked potentials (SEPs) and using transcranial magnetic stimulation (TMS) suggest that patients with focal dystonia have an abnormal processing of somatosensory information within the lemniscal system (for a review see Zeuner and Molloy, 2008). For example, patients with cervical dystonia revealed both abnormal cortical excitability and intracortical inhibition (Kanovsky et al., 2003). Further, the attenuation of SEPs seen in healthy people before and during hand movements is not observed in patients with writer's cramp indicating an impaired process of “sensory gating” in patients with focal hand dystonia (Murase et al., 2000). TMS protocols revealed long-afferent inhibition is selectively impaired in patients with focal hand dystonia: motor cortical excitability was not reduced 200–1000 ms after a conditioning stimulation of the contralateral median nerve and inhibition was converted into facilitation (Abbruzzese et al., 2001). Brain imaging data from dystonic guitarists during dystonia-inducing exercises revealed abnormal pattern of brain cortical activation characterized by an enhanced activation of the contralateral primary sensorimotor cortex and a bilateral underactivation of premotor areas (Pujol et al., 2000).

**Figure 2:**
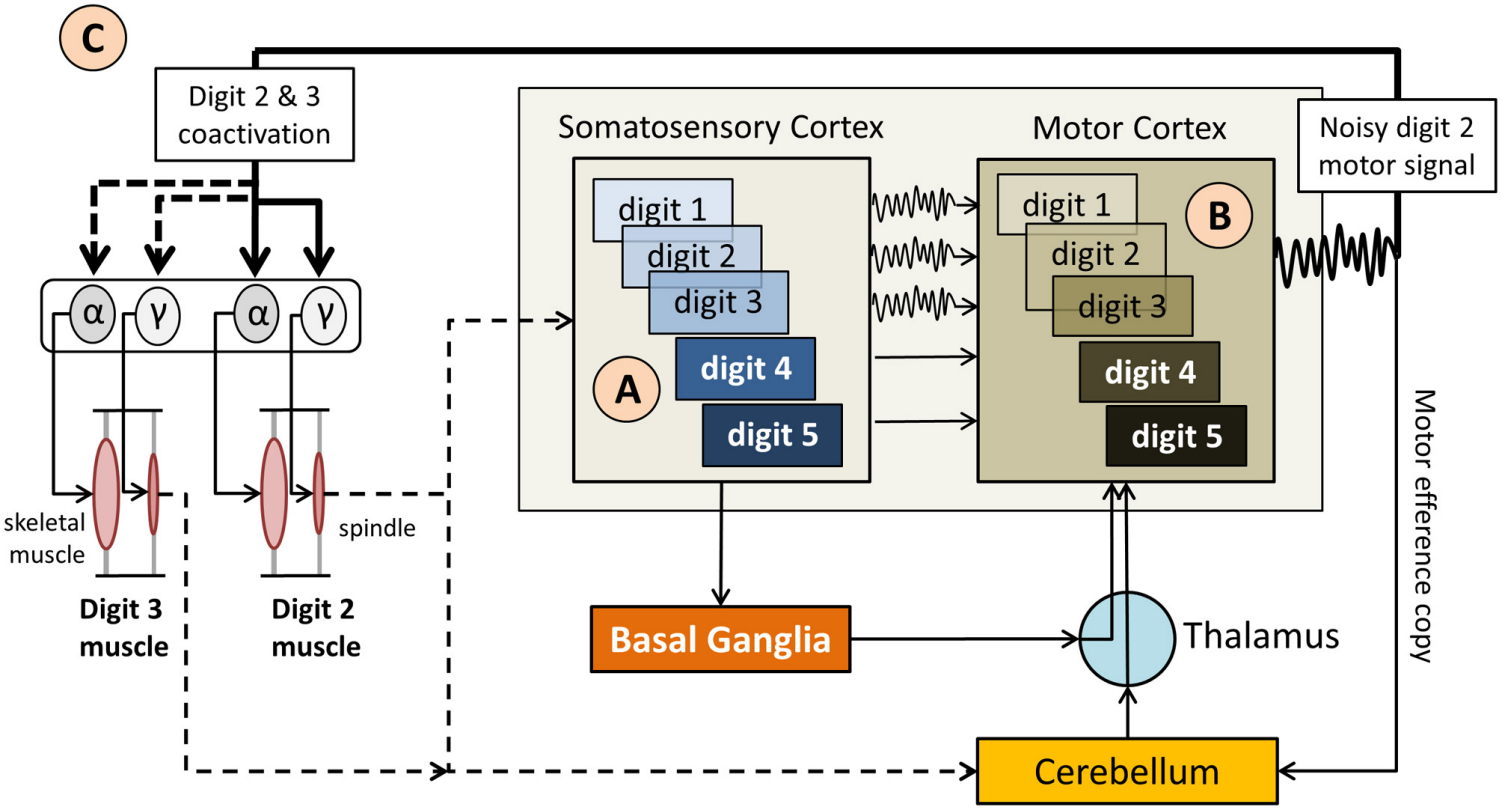
**Simplified diagram of the sensorimotor network in musician's dystonia. (A)** “Smeared” overlapping somatosensory representations of the hand digits (indicated as 1–5) provide imprecise, noisy feedback 
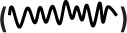
to the motor cortex, which also has overlapping motor representations of the digits. **(B)** The resulting motor signal originates from motor neurons representing multiple digits, i.e., are not finely tuned. **(C)**. As a result it comes to an involuntary coactivation of digits and faulty input to the γ motor neurons, which up-regulate spindle sensitivity. This, in turn, leads to abnormal proprioceptive feedback to the somatosensory cortex and the cerebellum. Not shown here is that the cerebellum modulates muscle tone based on propriospinal feedback, which further impacts volitional motor control.

Probably the most important neurophysiological correlates of focal dystonia are the enlarged and partially overlapping receptive fields in the somatosensory and motor cortices of patients with writer's cramp (Bara-Jimenez et al., 1998; Meunier et al., 2001) and MD (Elbert et al., 1998). This “smearing” of receptive fields mimic those seen in primate models of focal hand dystonia (Byl et al., 1996). It is believed that the lack of clearly defined somatosensory and motor cortical representations leads to the involuntary motor output seen in dystonia (see Figure 2). Abnormal somatosensory finger representations are not restricted to the dystonic regions, but have also been observed within the primary sensory cortex of the non-dystonic hand (Meunier et al., 2001). This corroborates the notion derived from behavioral findings that the dystonic motor symptoms are focal, but the underlying somatosensory dysfunction is general. That is, the sensory dysfunction can affect homologous and non-homologous somatosensory systems as demonstrated by reduced proprioceptive sensitivity of the contralateral hand in writer's cramp or a decreased arm position sense in patients with cervical dystonia or blepharospasm (Molloy et al., 2003; Putzki et al., 2006). Moreover, there is evidence that also the motor performance of non-dystonic limb systems may show subtle abnormalities. For example, patients with cervical dystonia show slightly abnormal kinematic features of their hand trajectories during reaching (Pelosin et al., 2009). If one considers further that long years of practice by professional musicians are associated with enlarged cortical representations in the somatosensory and auditory domains (Pantev et al., 2001) and focal dystonia is associated with overlapping receptive fields, it becomes plausible that musicians are especially susceptible to focal dystonia.

What emerges from the large body of neurophysiological research on focal dystonia is that MD and other types of task-specific dystonia are linked to the dysfunction of a complex network comprising basal-ganglia-thalamic-frontal cortex connections but also the inferior parietal cortex and the cerebellum (Avanzino and Abbruzzese, 2012). The notion of a basal ganglia involvement in the disease is further underlined by the fact that patients with Parkinson's disease, another disease affecting the basal ganglia, show similar deficits in haptic, tactile and kinesthetic perception (Maschke et al., 2003; Konczak et al., 2007, 2008, 2012). How the dysfunction of supraspinal circuitry affects the spinal motor neuron drive and how this interaction ultimately causes dystonic movement is still a matter of debate. We will discuss this linkage in more detail below when we outline the motor control behind MD.

## Mechanisms of somatosensory and sensorimotor integration

In order to understand the possible role of somatosensory processing in focal dystonia and specifically MD, it is important to understand how sensory and motor information are combined to produce movement, and to briefly review and define the underlying processes of multimodal sensory and sensorimotor integration.

The somatosensory system incorporates several sensory modalities namely touch, temperature, proprioception, and nociception (pain). These sensory modalities rely on signals from a wide variety of receptors embedded in the skin, muscles, tendons, and ligaments. With respect to motor behavior, touch and especially proprioception are known to be vitally important for reflexive and volitional motor control (Sainburg et al., 1995). The conscious perception of limb motion and position requires inputs from multiple receptors and the combined firing of various receptor types. It relies on responses from muscle spindles, Golgi tendon organs, and receptors in joint capsules. After the signals from these receptors enter the central nervous system (CNS), a cascading series of higher order neurons in the spinal cord, brainstem, cerebellum, basal ganglia, and neocortex process the available stream of proprioceptive information. Within the CNS proprioceptive signals will be combined with inputs from other sensory modalities in a process called *sensory integration* (for the purpose of this article we refer to all forms of combining sensory inputs as integration, recognizing that from a computational point of view integration can also be a specific mathematical operation performed by neural networks). In a second process of *sensorimotor integration*, sensory data are mapped onto volitional motor commands. In general, the term *sensorimotor integration* describes all processes where sensory information is used to plan and execute volitional movement.

While volitional motor commands give mostly rise to the *phasic innervation* of muscles, the interaction between proprioceptive afferents and descending commands at the spinal level shape patterns of *tonic innervation* that are vital for the control of muscle tone. Given that dystonia is considered a disease of abnormal muscle tone, it is worth noting that spinal motor neurons are specialized for the type of activity in which they normally participate. Animal research on crustaceans has shown that “tonic” motor neurons are responsible for maintaining activity by firing at moderate to high frequencies intermittently or continuously during locomotion and posture, while those recruited for short-lasting rapid responses (“phasic” neurons) generally fire a few impulses in a rapid burst during rapid locomotion and are otherwise silent (Millar and Atwood, 2004). At this point is not known, if the abnormal descending motor commands leading to dystonic postures preferentially target “tonic” spinal motor neurons.

However, neurophysiological research has investigated how supraspinal input affects spinal γ motor neuron output in focal dystonia. One paradigm to investigate this interaction is to apply muscle vibration. High frequency topical vibration of a skeletal muscle stimulates the Ia afferents of muscle spindles and is known to induce a tonic vibration reflex, a sustained muscular contraction. For example, if the biceps muscle is vibrated, a person's arm will extend. In addition, vibration alters the perception of forearm position and the actual arm position will be misjudged. Vibrating dystonic arm muscles of patients with writer's cramp can trigger dystonic postures (Kaji et al., 1995), while vibrating non-dystonic arm muscles in patients with cervical dystonia and blepharospasm will result in a more skewed arm position sense when compared to healthy controls (Grünewald et al., 1997). Given that the tonic vibration reflex is a spinally mediated reflex, these findings indicate that altering peripheral proprioceptive input affects proprioception and the regulation of muscle tone. However, knowing that peripheral receptors such as muscle spindles are spared in focal dystonia (Swash and Fox, 1976) the abnormal regulation of muscle tone is not explained as the result of faulty peripheral receptors. A more plausible explanation is that altered descending cortical signals excite both α and γ spinal motor neuron pools. Consequently, the increased γ motor neuron output resets muscle spindle sensitivity, which in turn will up-regulate muscle tone (see Figure 2).

## A computational approach to sensorimotor integration and motor control

**Figure 3:**
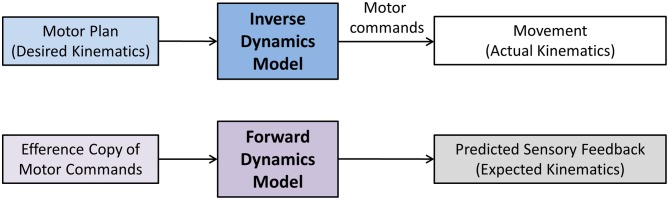
**Sensorimotor and motor-sensory transformations performed by inverse and forward dynamics models**. The term dynamics refers to Newtonian dynamics. That is, the transformation is between kinematics (movement) and kinetics (underlying forces). Here, a motor plan specifies the desired kinematics of the movement (desired trajectory, speed, acceleration). An inverse dynamics model transforms the planned kinematics into a stream of motor commands. These motor commands ultimately result in the appropriate generation of forces (kinetics) that will move the limb-body system in the desired way. A forward dynamics model essentially performs the reverse operation. Here, an efference copy of motor commands is used to predict the movement outcome.

**Figure 4:**
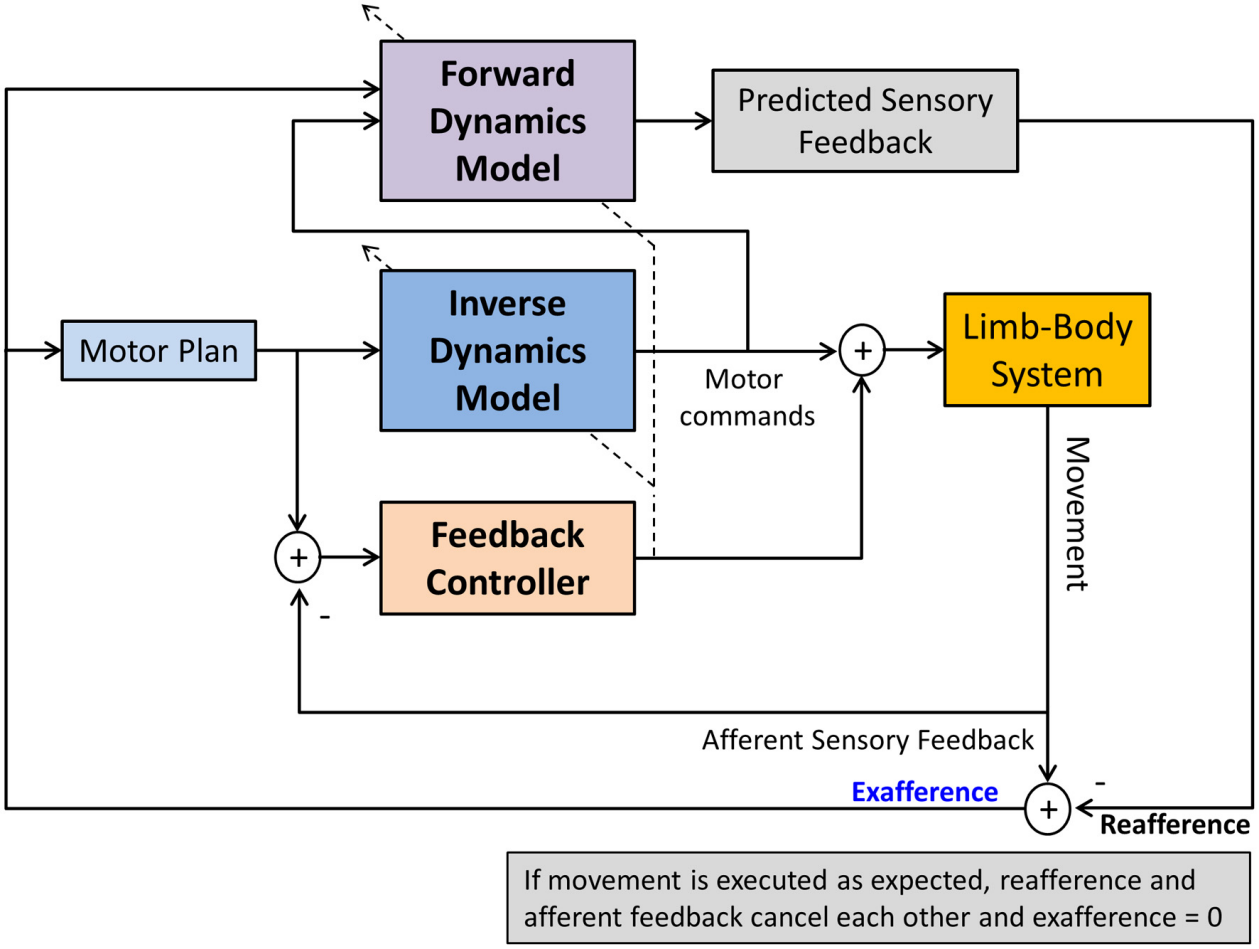
**A computational model of voluntary sensorimotor control**. Parallel feedback and feedforward mechanisms (inverse dynamics model) shape the motor commands. A feedforward dynamics model generates predicted (expected) sensory feedback based on the specified motor commands. Predicted and afferent sensory feedback are compared (reafference vs. afference) resulting in an exafferent signal. With respect to one's own movement, this signal indicates how “well” the movement was executed in relation to the plan. After a skill has been learnt, this mechanism can monitor the success of the execution, generating an error signal, if there is a discrepancy between desired and actual movement outcome. Such signal could be used to update the motor plan and/or to train the FDM. The dashed lines crossing the IDM and FDM indicate that both models can also be updated by afferent feedback in a process of sensorimotor adaptation.

The terms sensory integration and sensorimotor integration are often used to simply indicate that multimodal sensory information is combined with volitional motor commands. Unfortunately, such generic use of the terms is not necessarily helpful in discerning and identifying the exact operations underlying sensorimotor integration and control and consequently may have more of a metaphorical quality. Computational models of sensorimotor control seek to more clearly delineate what processes are necessary to achieve sensorimotor integration and to enable control. In general, a computational approach promotes that neural representations of the limb dynamics or kinematics, so-called *internal motor models*, form the basis of sensorimotor control. Two types of internal motor models can be distinguished. An *inverse dynamics model* (IDM) is part of a neural controller that transforms planned kinematic trajectories into appropriate patterns of muscular innervation (Kalveram, 1992; Wolpert et al., 1995; Konczak et al., 2003) necessary to generate the dynamics (i.e., the joint forces) to realize the planned movement (see Figure 3). A *forward dynamics model* (FDM) transforms efferent motor commands that specify limb dynamics into a set of joint kinematics (Wolpert and Kawato, 1998). In simple terms, an inverse model transforms the kinematic data of a movement plan into the necessary limb dynamics, while a forward model performs the opposite transformation, that is, it takes a set of joint dynamics as input and computes joint kinematics that would result from generating the specified dynamics. Both models transform sensorimotor data and are thus process modules of sensorimotor integration. Both are part of a voluntary motor control system were IDM, FDM and a feedback controller operate in parallel and the efferent motor signals reaching the muscles are shaped by the output of these three control systems (see Figure 4).

In addition, both model types are adaptable. That is, their model parameters can be changed by sensory input (see Figure 4). Proprioceptive information is known to be essential for the sensorimotor adaptation to biomechanical changes of the limb system or to changes in environmental forces (e.g., Coriolis or centripetal forces). For example, consider that movements such as reaching and grasping are controlled by a neural system resembling an IDM. The model parameters of the IDM represent neural estimates of specific biomechanical variables such as mass, gravity, stiffness and damping. Incorrect neural estimations of these biomechanical variables will lead to a loss of control and result in a motor deficit, such as hand trajectories becoming hypermetric, bradykinetic, or ataxic. Thus, the neural system is in constant need to update neural estimates of limb dynamics in order to maintain control. Any changes in the biomechanics of the limb system (such as additional forces acting upon the limb system) require either to update the existing model or the build a new model that captures the new dynamics. There is evidence that the cerebellum is critically involved in updating or building of new limb dynamics representations (Kawato et al., 2003) and that these representations become impaired in cerebellar disease (Maschke et al., 2004).

In contrast, forward models do not function as motor controllers but they augment motor control and perception. Input to a FDM is an efference copy of a motor command, the output is predicted sensory feedback (PSF) or the *reafference* (von Holst, 1954). An FDM thus performs an operation that may be considered as a process of motor-sensory integration, where the input is a movement related signal and the output is a sensory signal. In general, PSF signals can be used for a variety of sensorimotor functions. They help to improve haptic perception of objects by removing the contribution of the ego motion on the perception, and they are useful as control signals for very fast movements when the latency of afferent feedback is known to be too long to be useful for stable control (see Figure 4).

In the following we will adapt this outlined computational framework of sensorimotor control in order to establish a link between the observed sensory deficit and the motor disorder and to help us to understand the underlying mechanisms of sensory tricks in dystonia.

## Why do sensory tricks reduce symptoms in dystonia?

As we outlined above, substantial evidence exists showing that focal dystonia is associated with impaired processing of somatosensory information. It is further known that “sensory tricks” or “geste antagoniste” provides temporary relief of dystonic symptoms. These tricks usually involve altering tactile or proprioceptive feedback of the dystonic musculature. They are used by many patients and are well described in clinical literature (Poisson et al., 2012). For example, dystonic gestures during guitar playing may become less pronounced, if the playing hand wears a thin glove. In cervical dystonia, where the head is twisted toward the dystonic side touching the neck or chin on this side can induce the return to a normal head posture in some patients, with the effect persisting over several minutes. A recent report showed that the beneficial effect of sensory tricks is associated with better visuo-tactile discrimination and shorter disease duration (Kägi et al., 2013). Although its physiology is not fully understood, a brain imaging study of patients with cervical dystonia (Naumann et al., 2000) showed that the “sensory trick” reduces the activation of specific areas of the cortex (supplementary motor area and primary sensorimotor cortex) thus suggesting an abnormal central integration of sensory information. Two questions arise from the clinical observations and the related research: First, why does this maneuver work at all? And second, why does it not last?

**Figure 5:**
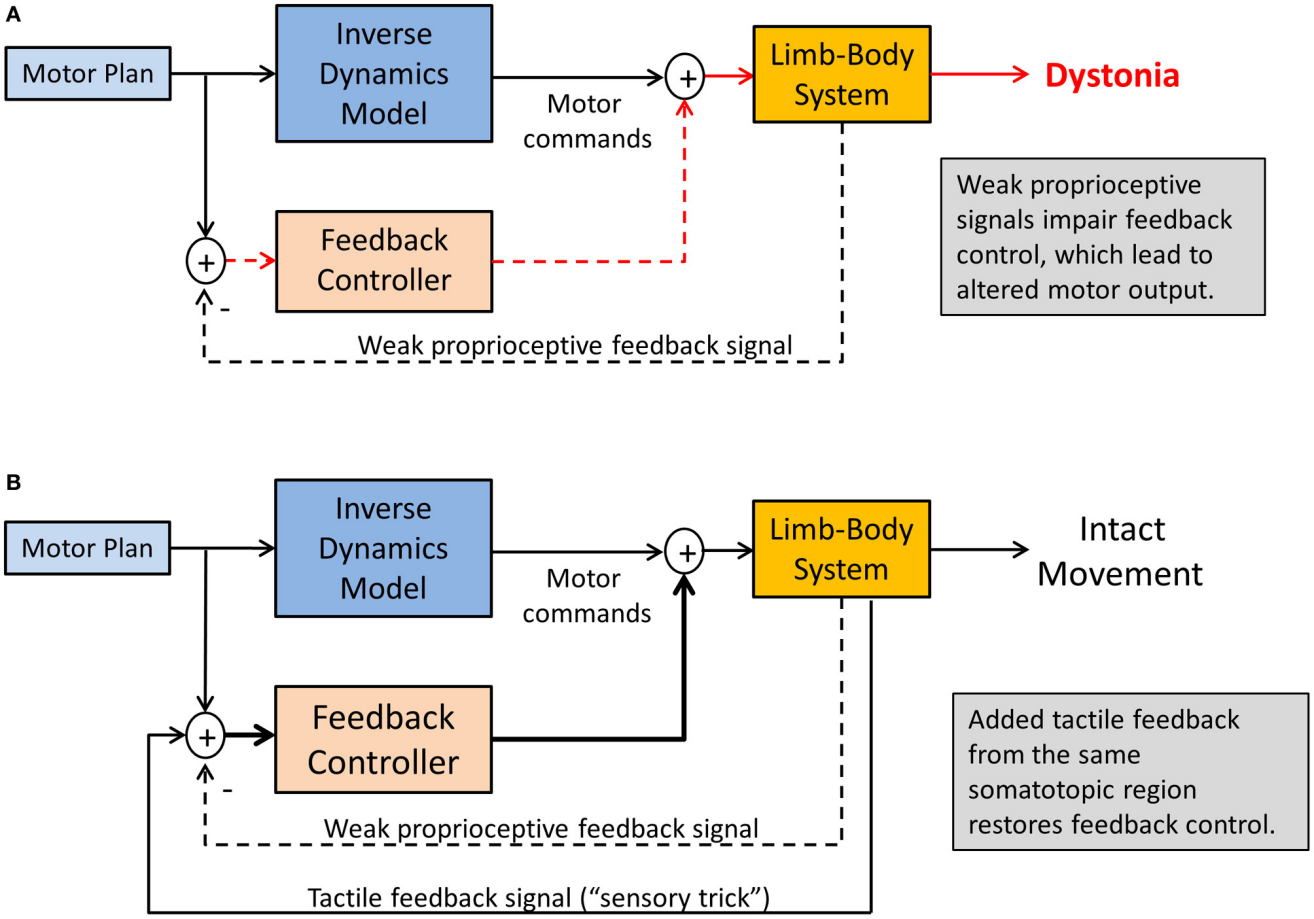
**Sensory tricks in dystonia**. Shown is a sensorimotor integration mechanism explaining the temporary relief from dystonic symptoms. **(A)** Weakened proprioceptive signals alters the feedback control for the tonic innervation of agonist-antagonist muscle groups, resulting in dystonic postures. **(B)** Tactile feedback related to the same somatotopic region of the dystonic musculature innervates multimodal tactile-proprioceptive neurons, essentially amplifying the proprioceptive signals. This will improve feedback control of muscle tone, resulting in symptom relief as long as tactile feedback is present.

There are two scenarios that may provide an answer. First, consider the abnormal head posture in cervical dystonia. Touching the affected dystonic neck or a region close to it provides a tactile stimulus related to the affected musculature, but it does not seem clear why such tactile signal carries any useful information that should help the motor system to improve head control and stabilization. In this case, it is likely imperative that the signal is somatosensory in nature and corresponds to the same somatotopic region as the dystonic musculature. Visual information or the touching of other body surfaces such as the opposite knee will not be helpful (Vacherot et al., 2007). It seems essential that the additional somatosensory stimulus activates similar or overlapping neural networks that process proprioceptive inputs within the same somatotopic regions of the somatosensory cortex and the basal ganglia. That is, the “trick” works, not because the touch information is informative for the head control system, but by amplifying proprioceptive signals through the concurrent recruitment of a related somatosensory sense, here touch (see Figure 5). This also implies that when the concurrent tactile stimulation stops, its motor effect will diminish relatively quickly.

Consistent with such explanation is that many neurons within the somatosensory cortices and the basal ganglia are known to respond to multimodal sensory stimulation (Lidsky et al., 1985; Ghazanfar and Schroeder, 2006). There is growing evidence that touch and proprioception are connected in both anatomy and function. For example, animal studies demonstrated that in the primary somatosensory cortex many neurons with cutaneous receptive fields encode elements both of tactile contact and self-motion (Rincon-Gonzalez et al., 2011). Functional imaging data in a study of kinesthetic illusions reveal that very similar cerebral networks including cortical and subcortical sensorimotor areas are activated for both touch and kinesthetic perception and that these networks are also classically found in passive or imagined movement tasks (Kavounoudias et al., 2008). The strongest kinesthetic illusions occurred under proprio-tactile co-stimulation and the associated brain area activations were distinct from those evidenced under unimodal tactile or proprioceptive stimulation: the inferior parietal lobule, the superior temporal sulcus, the insula-claustrum region, and the cerebellum. With respect to MD and other forms of focal dystonia this implies that the additional tactile stimulation during a “sensory trick” recruits these additional networks that are likely not affected by the disease.

**Figure 6:**
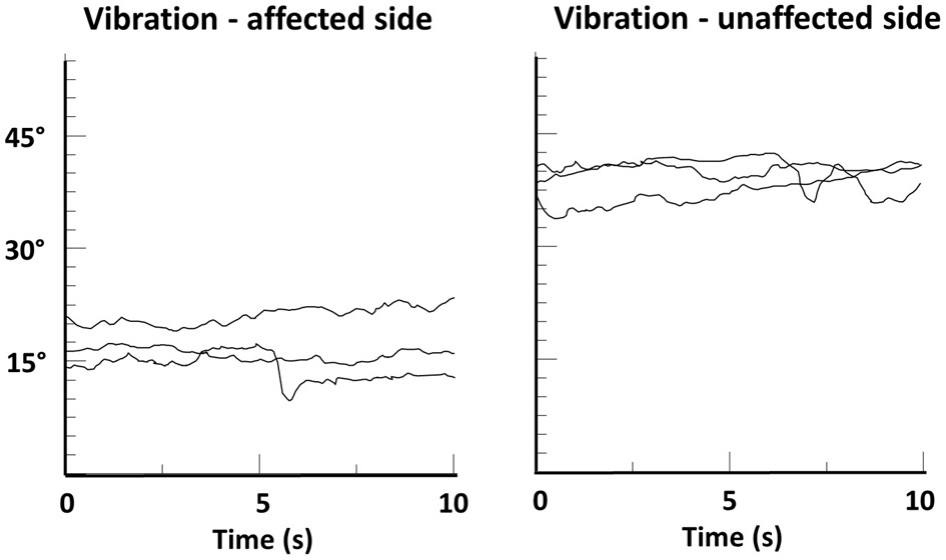
**Head position-time series data of a patient with spasmodic torticollis during muscle vibration of the left or right splenius muscle**. Shown are changes in head angle in the fontal plane during three trials of vibration, each lasting 10 s. A head angle of 0° corresponds to an upright head position. Note that head angle during vibration of the unaffected side remained above 30°, while vibration of the affected side resulted in a significant righting reaction (head angle ≈15°). From Karnath et al. (2000).

In addition, the importance of touch as an “amplifier” of proprioceptive processing is further underlined by the recent finding that patients with spasmodic torticollis who revealed a higher tactile precision at the finger were more likely to benefit from a tactile sensory trick than those with lower precision (Kägi et al., 2013). Furthermore, behavioral data from studying the *cutaneous rabbit effect* indicate that touch can be modulated by the integration of mechanical and proprioceptive input (Warren et al., 2011) (the effect describes a perceptual phenomenon where rapidly applied cutaneous stimuli induce the perception at a location where no stimulus was applied). Vice versa, there is recent evidence that concurrent tactile feedback enhances proprioceptive function in another basal-ganglia related disease. In a study with Parkinson's disease patients tactile feedback improved the proprioceptive accuracy in an arm-matching task (Rabin et al., 2010). There is also evidence that stimulating the proprioceptors of the dystonic musculature may overcome dystonic postures. In patients with spasmodic torticollis muscle vibration of affected neck muscles helped to restore normal head position, again indicating that increasing the strength of the relevant proprioceptive signals may be one way to alleviate dystonic symptoms (Karnath et al., 2000) (see Figure 6). However, such treatment may also have detrimental effects and increase symptoms (Kaji et al., 1995), showing the need for research on the correct “dosage” and location of such stimulation.

In a second scenario, consider the guitarist who uses a glove to overcome the task-specific dystonia in one or more digits of the plucking hand. Here the effect is a reduction in amplitude and frequency of dystonic finger motion improving the player's musical performance. This effect is difficult to explain as a “sensory signal amplification” effect. A possible explanation relates to a fundamental mechanism of the sensorimotor system that compares afferent with PSF. In simple terms, afferent sensory feedback from the fingers touching the strings provides state information (“how does the movement feel”), while PSF provides information on the desired state (“how should it feel”). In our example, the glove substantially alters the exafference (“it does feel different”), while the PSF was unaltered. That is, there is an initial mismatch between actual and expected sensory feedback. Typically such mismatch indicates an error signal that, if large enough, would trigger a motor learning process. As the somatosensory feedback associated with the skilled movement is altered and the PSF no longer corresponds to it, the underlying FDM needs to adapt based on the new tactile feedback. That is, the altered afferent feedback triggers a process of where the old FDM gets updated, or a new FDM gets established. Consequently, the sensorimotor system goes through a process of motor-sensory adaptation and builds or updates the respective FDM associated with playing the guitar (the FDM could even be specific to a certain piece of music that is part of the player's acquired repertoire). Assuming that this adaptation process is intact in dystonia the mismatch between expected and state feedback will vanish over time. At that point the glove as an instrument of sensory alteration is no longer effective. The symptoms will start again and a new sensory trick will have to be found. In essence, this scenario describes a process of updating the *reafference*, that is, the expected sensory environment (auditory, tactile, proprioceptive) associated with the playing of the instrument (see Figure 4).

## Sensory training and sensory reorganization to overcome MD

Treatment with botulinum toxin (BT) can transiently modify the enlarged cortical maps seen in focal dystonia (Thickbroom et al., 2003) possibly by due to BT induced changes to the innervation of intrafusal muscle fibers (Trompetto et al., 2006). Such change in spindle sensitivity alters proprioceptive afferent signals that, in turn, indirectly promote cortical plasticity. It has been documented that such plastic changes are not restricted to somatosensory or motor cortical representations of the dystonic musculature itself. For example, when patients with cervical dystonia received BT injections into the cervical muscles their abnormal reaching kinematics of non-dystonic arms partially disappeared three weeks after injection, indicating a motor reorganization of clinically not affected body segments (Pelosin et al., 2009). Similarly, BT injected into neck muscles of cervical dystonia patients decreased sensorimotor associative plasticity in the hand area by reducing afferent input from neck muscles (Kojovic et al., 2011). While the primary treatment effect of BT by blocking the motor efferent signals at the motor endplates are well described, the secondary effect of restoring cortical maps has received far less attention. However, it indicates that BT, in some respect, may be regarded as a “drastic” form of a sensory trick (Rosales and Dressler, 2010), i.e., an intervention that alters the operation of the sensorimotor loop (see Figure 2).

In addition to BT therapy, behavioral training interventions that seek to modulate somatosensory input have gained prominence. There is evidence that sensory-motor retuning via splinting or through constraint-induced movement therapy does remodel cortical networks and may induce long-term reductions in the symptoms of focal hand dystonia (Candia et al., 1999). Magnetoencephalography confirmed that sensory-motor retuning modified cortical finger representations of the dystonic hand in such a way that it more closely resembled the organization of the non-affected side (Candia et al., 2005). Another study on 144 MD patients reported that 50% of patients benefitted from some form of behavioral or pedagogical training (Jabusch et al., 2005). In summary, these studies suggest that focal dystonia, in general, and MD specifically can be reversed by context-specific training protocols. The spread of symptoms might be prevented by avoiding training of movement patterns similar to the main affected task, and by reducing the amount of task-associated movement behavior (Candia et al., 2005).

## Conclusion

The motor symptoms of MD and various forms of focal and task-specific dystonia have been well described. In addition, research in the last two decades has provided solid evidence that patients with focal dystonia show deficits in somatosensory perception. We here presented a framework that links the observed somatosensory deficits to the observed motor symptoms in dystonia. According to this framework, the root of the dystonic postures is a somatotopically defined proprioceptive deficit that ultimately modulates the tonic and phasic innervation of those muscles within the affected somatotopic region. The neural basis of this somatosensory deficit likely relates to abnormal processing within the cortico-basal loop, which is known to be involved in the complex processes of sensory and sensorimotor integration. While the exact pathomechanism is not fully understood, the motor symptoms can be understood as a failure of cortical feedback control. Because feedback control of volitional movements closely interacts with feedforward control mechanisms, such loss of control leads to erratic failures during the execution of voluntary movements when feedforward motor commands trigger phasic innervation patterns. That is, although MD is a task-specific disorder, it is not sufficient to contend that the failure to produce a normal movement pattern is a sign of a dysfunction in “motor programming” or movement execution. Clearly, movement execution is affected, but the underlying causes are not explained by a deficit in motor planning or motor execution. They arise from a complex interaction of networks within the sensorimotor loop (see Figure 2). Tactile sensory tricks are known to temporarily alleviate dystonic motor symptoms. As we described, they “work” for at least two reasons: First, the mechanisms of proprio-tactile integration are still intact. Thus, combining the two related sensory streams results in enhanced signals that cortical feedback mechanisms can use to control tonic innervation (see Figure 5). Second, altering the somatosensory experience associated with a learnt skill will trigger adaptive processes of implicit motor learning, which also are likely intact in focal dystonia. In essence, the sensorimotor system utilizes a fully functioning motor efference copy mechanism to align experienced with expected sensory feedback. Computationally, this process is consistent with updating or the building of a feedforward model.

As a final remark we put forward that an enhanced knowledge about the underlying failure in sensorimotor control may not be helpful in the search for a cure of the disease. However, we contend it may be quite useful in designing behavioral therapies aimed at intervening or exploiting mechanisms of sensorimotor integration and control.

### Conflict of interest statement

The authors declare that the research was conducted in the absence of any commercial or financial relationships that could be construed as a potential conflict of interest.
